# Evidence That *STK19* Is Not an NRAS-dependent Melanoma Driver

**DOI:** 10.1016/j.cell.2020.04.014

**Published:** 2020-06-11

**Authors:** Marta Rodríguez-Martínez, Thierry Boissiére, Melvin Noe Gonzalez, Kevin Litchfield, Richard Mitter, Jane Walker, Svend Kjœr, Mohamed Ismail, Julian Downward, Charles Swanton, Jesper Q. Svejstrup

**Affiliations:** 1 Mechanisms of Transcription Laboratory, The Francis Crick Institute, 1 Midland Road, London NW1 1AT, UK; 2 Cancer Evolution and Genome Instability Laboratory, The Francis Crick Institute, 1 Midland Road, London NW1 1AT, UK; 3 Bioinformatics and Biostatistics, The Francis Crick Institute, 1 Midland Road, London NW1 1AT, UK; 4 Structural Biology Science Technology Platform, The Francis Crick Institute, 1 Midland Road, London NW1 1AT, UK; 5 Oncogene Biology Laboratory, The Francis Crick Institute, 1 Midland Road, London NW1 1AT, UK

## Abstract

*STK19* was proposed to be a cancer driver, and recent work by Yin et al. (2019) in *Cell* suggested that the frequently recurring STK19 D89N substitution represents a gain-of-function change, allowing increased phosphorylation of NRAS to enhance melanocyte transformation. Here we show that the *STK19* gene has been incorrectly annotated, and that the expressed protein is 110 amino acids shorter than indicated by current databases. The “cancer driving” *STK19* D89N substitution is thus outside the coding region. We also fail to detect evidence of the mutation affecting *STK19* expression; instead, it is a UV signature mutation, found in the promoter of other genes as well. Furthermore, STK19 is exclusively nuclear and chromatin-associated, while no evidence for it being a kinase was found. The data in this Matters Arising article raise fundamental questions about the recently proposed role for STK19 in melanoma progression via a function as an NRAS kinase, suggested by Yin et al. (2019) in *Cell*. See also the response by Yin et al. (2020), published in this issue.

## Introduction

Analysis of large-scale exome data led to the identification of *STK19* as a potential cancer driver gene, which harbors somatic hotspot mutations in melanoma (Hodis et al., 2012) and skin basal cell carcinoma (Bonilla et al., 2016). *STK19* is also listed among the top melanoma driver genes (Lawrence et al., 2014). These studies specifically annotated an *STK19* mutation (a C to T transition) causing a change at annotated amino acid 89 from aspartic acid to asparagine (D89N) as the melanoma driver. However, the mechanism underlying transformation to malignancy was unknown. A study by Yin et al. (2019) in *Cell* recently proposed that STK19 functions as an NRAS-activating kinase and that D89N represents a gain-of-function change, which increases STK19-mediated NRAS phosphorylation, thereby increasing the malignancy of NRAS-mutated melanomas (Yin et al., 2019).

We discovered STK19 in a multi-omic screening approach designed to uncover factors with a role in the cellular response to UV-generated DNA damage (Boeing et al., 2016). Given that it had previously been suggested that STK19 is a protein kinase, and that *STK19* had been uncovered as a melanoma driver, this was potentially extremely interesting. However, it soon became evident to us that much of the information on the *STK19* gene and its annotated protein product is mistaken. Here we present the evidence indicating that the *STK19* gene has been incorrectly annotated, with the expressed gene-product being 110 amino acids shorter than indicated by current databases, so that the only product of note is a protein of 29 kDa, not 41 kDa. Indeed, the “D89N” mutation is neither a coding mutation nor a melanoma driver, and STK19 is a nuclear, DNA-binding protein, which does not appear likely to be a kinase. In light of these findings, we suggest that the conclusions on *STK19* reported by Yin et al. (2019) need to be reconsidered.

## Results

### A 41 kDa Isoform of STK19 Protein Does Not Exist

**Figure 1:**
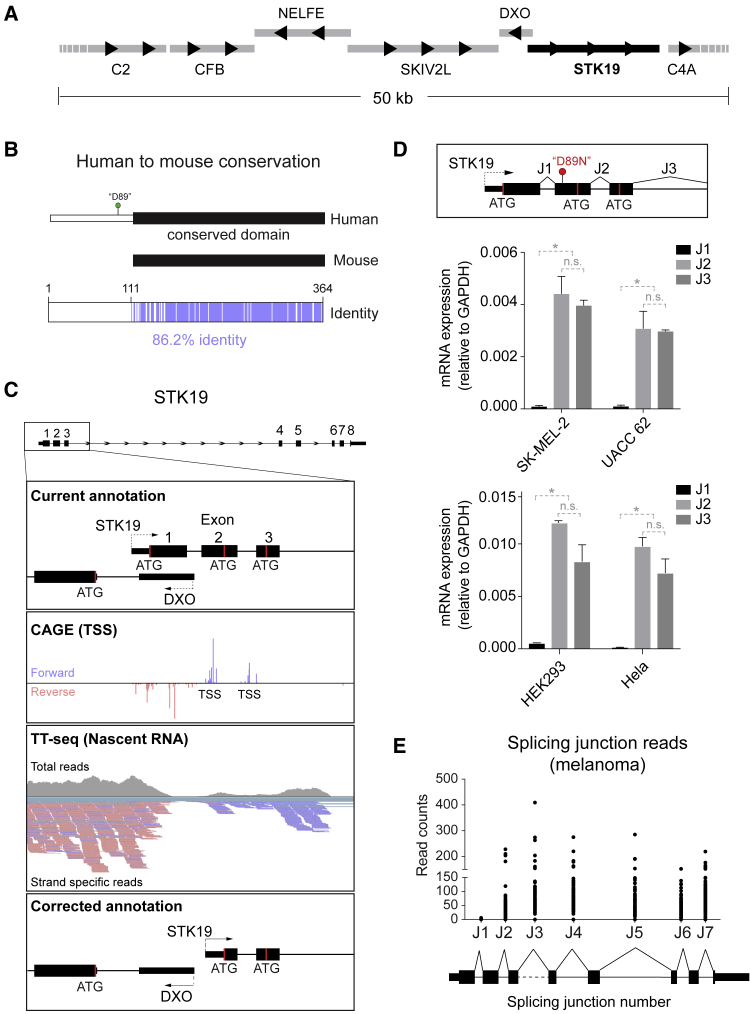
Correcting *STK19* Gene Annotation (A) Schematic representation of the gene-dense region around *STK19*, drawn to scale. Black arrowheads indicate direction of transcription. (B) Schematic representation of mouse and human STK19 protein homology, as the proteins are currently annotated. Black bars indicate what is being annotated as a conserved domain, whereas the white bar indicates a domain supposedly present only in the human isoform. Below, purple bars indicate amino acid identity (see also Figure S2). The position of amino acid D89 in the human STK19 is also indicated. (C) Diagram of the 5′ region of STK19 gene aligned to (top to bottom) CAGE (TSS) data from the FANTOM project, TT-seq data from HEK293 cells, and the proposed, corrected *STK19* 5′ annotation. Reverse strand reads are in pink, and forward strand reads are purple. (D) mRNA qPCR data on *STK19* splice junctions 1 (J1), 2 (J2) and 3 (J3). Splice junction numbers refer to the current *STK19* annotation shown above. Graphs show expression relative to GAPDH. Error bars represent ± SD. Statistically significant differences (p < 0.05, multiple t tests, Holm-Sidak correction) of three replicates are indicated with asterisks. Non-significant differences are indicated with “n.s.” when relevant. J1 is only detected at background level. (E**)** Splicing junction reads found in melanoma patient samples (n = 81). Splice junction numbers refer to the current STK19 annotation (see D.).

**Figure S1:**
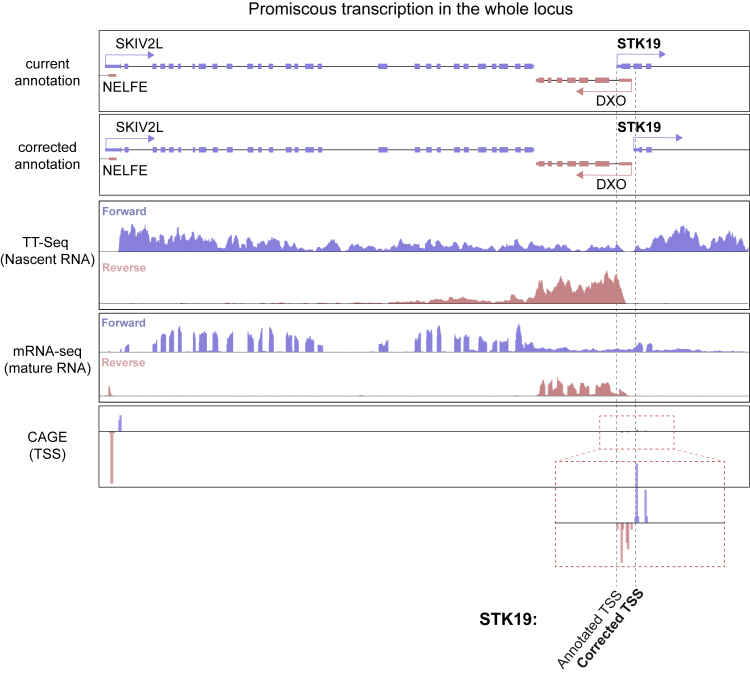
Promiscuous Transcription in the *STK19* Gene Locus, Related to Figure 1 *STK19* and relevant surrounding genes are shown. Forward strand genes are shown in blue (*SKIV2L* and *STK19*) and reverse strand genes are in pink (*NELFE* and *DXO*). The current annotation is aligned with the corrected annotation, TT-seq and mRNA-seq data are from WT HEK293 cells (Gregersen et al., 2019), and CAGE data from the FANTOM project to show TSSs. Red dashed box shows zoom-in of CAGE data of the *STK19* promoter region. Annotated TSS and corrected TSS are marked by dashed black lines.

The paper by Yin et al. (2019) is entirely focused on the study a 41 kDa STK19 isoform and its effect on NRAS activation. Indeed, western blots showing this 41kDa isoform are found throughout the paper, and almost all conceptually important experiments are based on its existence as the main form of STK19. The idea that STK19 is a 41 kDa protein originates in its initial annotation 30 years ago, and given the complexity of the locus in which the gene is located as well as the tools available at that time, mistakes are understandable. MHC III is the most gene-dense locus in the human genome (Xie et al., 2003), with the region around *STK19* being particularly compact (Figure 1A). Near *STK19*, *NELFE* and *DXO* are located on the reverse strand, while *SKIV2L*, *STK19* itself, and *C4A* are on the forward strand. The *DXO* gene is located between *SKIV2L* and *STK19*, but is so short (∼2.4 kb in total) that transcriptional readthrough from the upstream *SKIV2L* gene results in some mRNA from this gene being detected up to the beginning of *STK19* (Figure S1), underscoring the challenge in correctly annotating the 5′ end of the *STK19* gene, even with the detail provided by genome browsers today.

**Figure S2:**
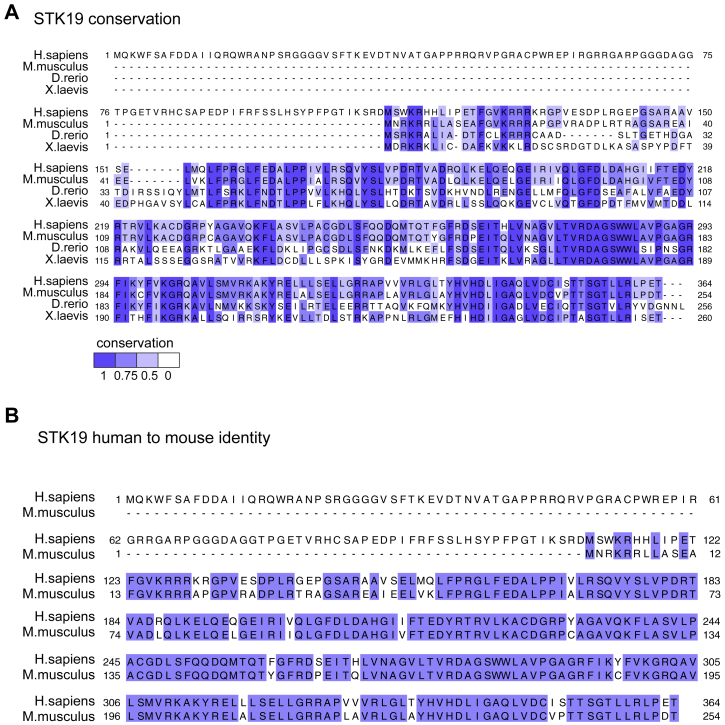
STK19 Protein Conservation, Related to Figure 1 (A) Alignment of the presently annotated STK19 protein from four different metazoans. Conservation ratio is shown as shades of purple as indicated in the legend below. (B) Alignment of mouse (*Mus musculus*) and the presently annotated human (*Homo sapiens*) STK19 proteins. Purple indicates identical residues.

A first, strong indication that the human *STK19* gene is mis-annotated is provided by the fact that even though the STK19 protein is highly conserved in vertebrates, the first 110 amino acids are not. If the present annotation were correct, only the hominid STK19 proteins contain this N-terminal 110 amino acid region (Figure S2A). Indeed, the human and mouse proteins are almost identical, except for the N-terminal 110 amino acids, which are entirely absent in the mouse (Figure 1B and S2B). These findings are of particular relevance given that several experiments in Yin et al. (2019) were performed with the human 41 kDa protein in a mouse model system.

**Figure S3:**
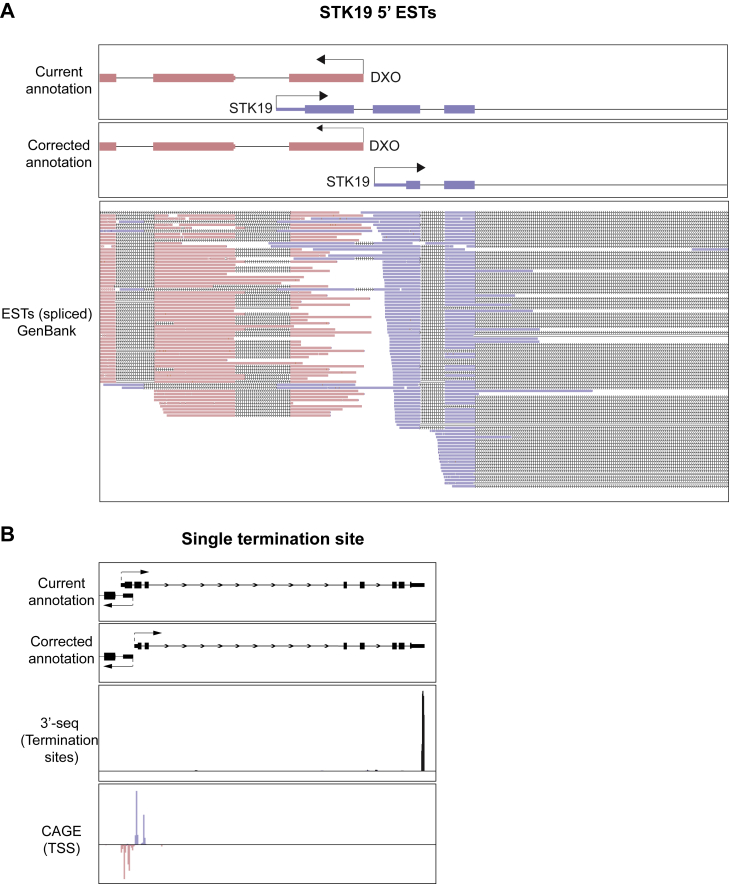
*STK19* ESTs and Termination Site, Related to Figure 1 (A) Genome browser view of *STK19*’s 5′ region. Current annotation and corrected annotation are aligned to spliced GeneBank ESTs. The reverse strand gene (*DXO*) and ESTs are shown in pink, and forward gene (*STK19*) and ESTs are shown in blue. The few ESTs upstream of the corrected annotation likely originate from transcriptional readthrough of *SKIV2L* or from promiscuous transcription of the locus. (B) Genome browser view of *STK19*, with current and corrected annotations shown aligned to transcription termination sites, mapped by 3′-seq in HEK293 cells (Gregersen et al., 2019), and transcription start sites (TSSs) mapped by CAGE by in the FANTOM database. Forward TSS are shown in blue and positive and reverse TSS in pink and negative.

Further evidence of mis-annotation comes from the transcription start sites (TSSs) mapped by CAGE (cap analysis of gene expression) as part of the FANTOM project (Abugessaisa et al., 2017, Bertin et al., 2017). These data indicate that the TSS for the human *STK19* gene resides in the second exon, and that another, much weaker TSS may exist in the third exon of the gene as currently annotated (Figure 1C, CAGE). Nascent transcription measured by TT-seq in HEK293 cells (Gregersen et al., 2019) further supports the idea that the main, most upstream *STK19* TSS resides in what is presently annotated as the second exon (Figure 1C, TT-Seq). We note that these observations are also in agreement with the expressed sequence tags (ESTs) found for *STK19* in the GenBank database, in which transcripts are only consistently detected from those start sites (Figure S3A). These data alone suggest that *STK19* gene annotation should be altered, so that the correct beginning of the STK19 transcript is located in what is presently annotated as the second exon (Figure 1C, corrected annotation at bottom; compare to current annotation in upper panel). The correct first amino acid is thus methionine 111 in the current protein annotation, as is also suggested by the protein conservation data (Figure 1B).

In light of the data reported by Yin et al. (2019), we checked whether the melanoma cell lines used in their study might show a different mRNA isoform expression from those described above. For this purpose, we used splicing of the first exon-exon junction (J1) of the current annotation as a readout, with junctions 2 and 3 (J2 and J3) as controls (see schematic in Figure 1D, upper panel). Given that Yin et al. (2019) detected a 41 kDa protein in these cells, the encoding mRNA isoform, containing J1, must obviously be expressed. However, in two different melanoma cell lines used by Yin et al. (SK-MEL-2 and UACC 62), as well as two commonly used human cell lines (HEK293 and HeLa), J1 was not detected above background level by quantitative PCR of *STK19* cDNA, while J2 and J3 were clearly detected (Figure 1D), consistent with the actual TSS residing in the 2^nd^ exon of the annotated STK19 gene.

We also tested if expression of the annotated transcript might be specific to melanoma samples by analyzing the relative abundance of J1 reads compared to reads from the other splice junctions in the transcript (J2 to J7) using melanoma RNA-seq data from 81 patients. As shown in Figure 1E, J1 is detected only at background levels, whereas all other splice junctions are detected at similar, much higher levels. Together, these data indicate that the 5′ region of *STK19* gene is presently mis-annotated, with the actual TSS located markedly downstream (∼490 bp) from the currently annotated TSS. These data indicate that the annotated 41 kDa STK19 isoform is not expressed as its initiation codon lies outside of the actual *STK19* gene.

**Figure 2:**
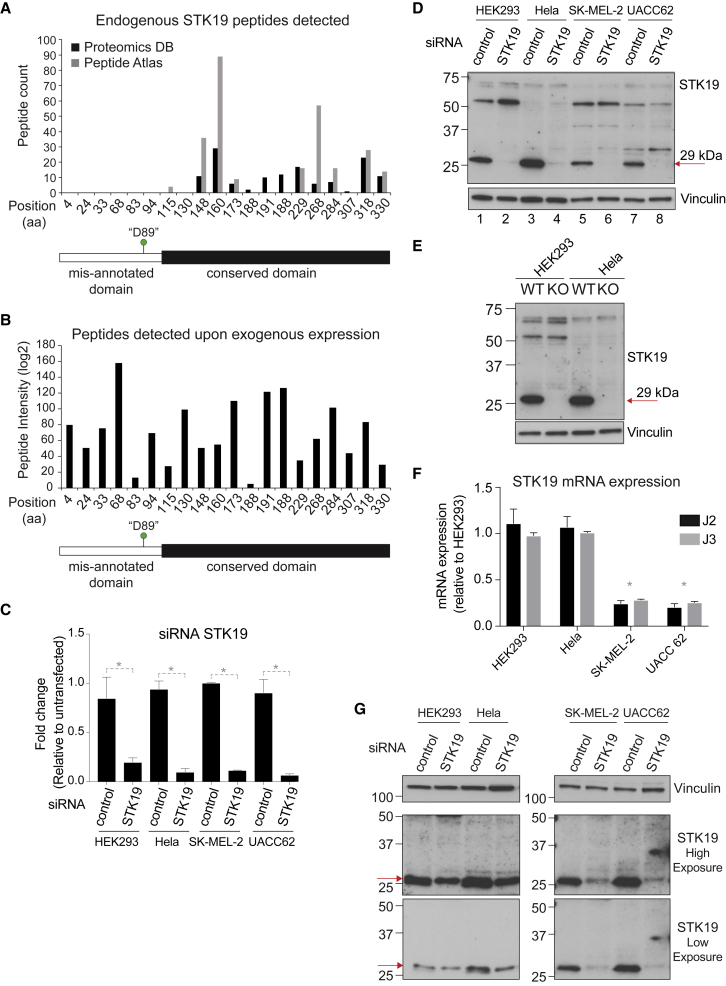
Correcting STK19 Protein Annotation (A) Graph showing STK19 peptide counts from Proteomics BD and Peptide Atlas, aligned to the amino acid position in STK19, above a schematic representation of STK19 as currently annotated showing the conserved domain, the miss-annotated domain and the position of “D89” mutation. (B) As in (A), but showing average peptide intensity found by mass spectrometry analysis of overexpressed, immunoprecipitated 41 kDa STK19 protein. (C and D) siRNA of STK19 in HEK293, HeLa, and melanoma cell lines SK-MEL-2 and UACC 62. C, Graph showing the *STK19* mRNA expression relative to control. ΔCT values were calculated relative to GAPDH before comparing to control. Error bars represent ± SD. Statistically significant differences (p < 0.05, multiple t tests, Holm-Sidak correction) of three replicates are indicated with asterisks. (D) Western blot of STK19. Vinculin is used as loading control. 75 μg of whole cell extract were loaded per lane. Arrow on the right indicates the only specific STK19 band detected, corresponding to the 29 kDa protein. (E) Western blot of whole cell extract of *STK19* wildtype and knock-out cell lines (HEK293 and HeLa). 75 μg of total whole cell extract loaded per lane. Arrow on left indicates the only band that disappears after gene knockout, the 29kDa species. (F) qPCR data of STK19 mRNA expression in the cells used in (C and D). ΔCT values were calculated relative to GAPDH before normalizing to the expression level in HEK293. Error bars represent ± SD. Asterisks indicate statistically significant differences (p < 0.05, multiple t tests, Holm-Sidak correction) of three replicates of HeLa or 293 compared to SK-MEL-2 and UACC 62 for both J1 and J2. (G) Western blot of STK19 in extracts from HEK293, HeLa, and melanoma cell lines SK-MEL-2 and UACC 62, following the protocols provided by Yin et al. 2 different exposures are shown. Red arrow indicates the position of STK19; no band is visible at 41 kDa. siRNA of STK19 was less efficient than in 2D.

Nevertheless, in a further effort to investigate whether an endogenous 41 kDa STK19 isoform might exist, we first analyzed the STK19 peptides found in the large proteomics database Proteomics BD (Schmidt et al., 2018) and in the Peptide Atlas database (Deutsch et al., 2008). The latter database provides a unique tool for targeted proteomics as it only accepts raw data that is analyzed so that specific peptides can be reliably quantified across independent experiments. Importantly, neither database contains reliable, unique peptides mapping to the first 110 amino acids of the currently annotated 41 kDa STK19 protein, with the first peptide detected starting at amino acid 115 of the currently annotated form (Figure 2A), as expected if trypsin cuts STK19 on the C-terminal side of lysine 114 (i.e., lysine 4 in the corrected annotation). It could be argued that a lack of detection by mass spectrometry of peptides over a certain region might merely be due to the characteristics of the tryptic peptides originating from that region, i.e., that they are intrinsically difficult to detect. We therefore performed proteomic analysis of STK19 using HEK293 cells expressing a doxycycline-inducible, transgene encoding a flag-tagged 41 kDa isoform protein and found that peptides from the first 110 aa of the annotated STK19 protein *can* be detected when it is exogenously expressed at four different levels of expression (Figure 2B). The lack of peptide detection from this region in the protein databases further indicates that this region is simply not encoded and thus not part of the endogenous STK19 protein.

To follow up on these findings, we performed western blot analysis in four different cell lines, two of which were used by Yin et al. and showed endogenous STK19 as a 41 kDa protein. In an effort to faithfully reproduce their results, we used the same STK19 antibody as Yin et al. (NBP2-33955, Novus Biologicals). To assess antibody specificity, siRNA-mediated STK19 knockdown was performed, which was efficient, as detected by qPCR (Figure 2C) and western blot analysis (Figure 2D, red arrow; lanes 2, 4, 6, and 8). Western blot analysis of these samples showed various non-specific bands that were unaffected by STK19 siRNA knockdown, while the only specific STK19 band migrated at 29 kDa (Figure 2D). This result was further confirmed in *STK19* CRISPR knockout (KO) cell lines (Figure 2E). Note that STK19 protein levels in HeLa and HEK293 cells are somewhat elevated compared to the melanoma cell lines, which correlates with relatively higher mRNA expression in these cells (compare Figure 2F and 2D).

These results are in agreement with the predictions from gene expression- and proteomics studies, but inconsistent with those reported by Yin et al. Therefore, in order to test whether the diverging western blot results might be due to a difference in experimental approach, we obtained the cell extraction and western blot protocols used in Yin et al. from the Cui laboratory and repeated the analysis using their protocol. Again, we only detected a 29 kDa isoform (Figure 2G, red arrows), with no detectable 41 kDa protein in the human cell lines used by Yin et al. (2019).

**Figure 3:**
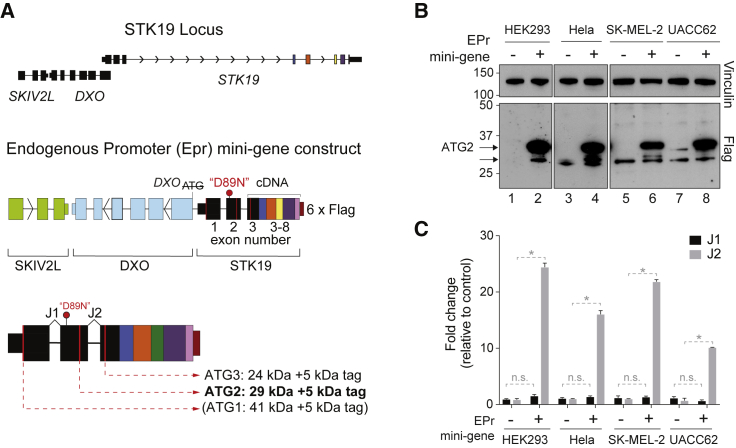
Multi-copy Overexpression of STK19 from Its Endogenous Promoter (A) Schematic representation of the endogenous promoter (EPr) system created to assess STK19 protein isoform expression. The upstream region of *STK19*, encompassing the end of the *SKIV2L* gene and the entire *DXO* gene, as well as the first 2 exons and 2 introns (following the current annotation) were fused with cDNA encoding exons 3 to 8. Since the *STK19* promoter may overlap with the upstream *DXO* gene, the ATG of this gene was mutated to avoid DXO protein expression. Sequence encoding a 2x triple-FLAG tag was inserted at the 3′ end of the construct. Exon numbers and the location of the “D89N” mutation are shown. Schematic representation of STK19 isoforms that could conceivable be expressed are shown below. (B) Western blot of whole cell extract of the transient transfection of the EPr construct, compared to empty vector in the indicated cell lines. Arrows indicate the expressed isoforms. Bands at 34 and 29 kDa correspond to expression from the 2^nd^ and 3^rd^ ATG (or possibly a degradation product), respectively. (C) qPCR of *STK19* expression after transient EPr or empty vector transfection (relative to endogenous untransfected control and normalized to GAPDH levels) comparing exon-exon junction 1 (J1) and 2 (J2) as per the current annotation. Error bars represent ± SD. Statistically significant differences (p < 0.05, multiple t tests, Holm-Sidak correction) of three replicates are indicated with asterisks. Non-significant differences are indicated with “n.s.” when relevant.

*STK19* is very lowly expressed and we have only been able to detect the endogenous form with the antibody from Novus Biologicals. We took our study one step further to investigate whether a 41 kDa isoform might be expressed and detected when using a STK19-encoding construct containing the 5′ regulatory region, the first three exons with their intervening introns, followed by a cDNA fusion of the remaining, uncontroversial exons of the *STK19* gene (Figure 3A). 3′-seq data (Gregersen et al., 2019) had previously identified a single, unique *STK19* transcription termination site (TTS) (Figure S3B), making it straightforward to correctly place a 2x triple-Flag tag after the final amino acid of STK19. This *STK19* mini-gene, which contains parts of the *SKIV2L* coding region and the entire *DXO* locus (with its ATG mutated), thus has *STK19* expressed from its own endogenous promoter (EPr) and maintains normal *STK19* regulation, including splicing across the first three exons relevant for isoform expression. Importantly, it enables visualization of any isoform that might not have been detectable due to the low level of endogenous *STK19* expression and the difficulty in detecting the encoded protein with anti-STK19 antibodies. The EPr mini-gene construct was overexpressed by transient transfection into the four different cell lines previously used (including two used by Yin et al.), and the resulting samples were analyzed by western blotting (Figure 3B) and RT qPCR (Figure 3C). Again, no evidence for a protein corresponding to the annotated 41 kDa protein isoform was observed. Rather, the slowest migrating, specific band detected by anti-FLAG antibody corresponds to the 29 kDa isoform (which migrates at 34 kDa due to the 2x triple-Flag tag) (Figure 3B, lanes 2, 4, 6, and 8). A weaker, slightly faster migrating band, corresponding either to expression from the downstream ATG3 or possibly a degradation product, was also detected. Importantly, qPCR analysis further showed that whereas splice junction 2 (J2) increased ∼25-fold compared to the endogenous gene in the same cells, expression of J1 did not increase after overexpression (Figure 3C; see graphic representation in 3A, lower panel). This further supports our data that J1 is not expressed and thus that the annotated 41 kDa STK19 protein isoform is not produced.

### The “D89N” Mutation Is Not in the Coding Region and Does Not Affect Protein- or Gene-Expression

Whether a 41 kDa STK19 isoform exists or not is crucial as the amino acid alteration in STK19 D89N reportedly represents a cancer-driving change, which would not be encoded in the 29 kDa STK19 protein described here (see Figure 3A, lower panel). Indeed, Yin et al. (2019) compared the (41 kDa) STK19 D89N protein with the wild type counterpart and expressed the mutant in both human cells and in mice treated with an STK19-directed small molecule inhibitor.

**Figure 4:**
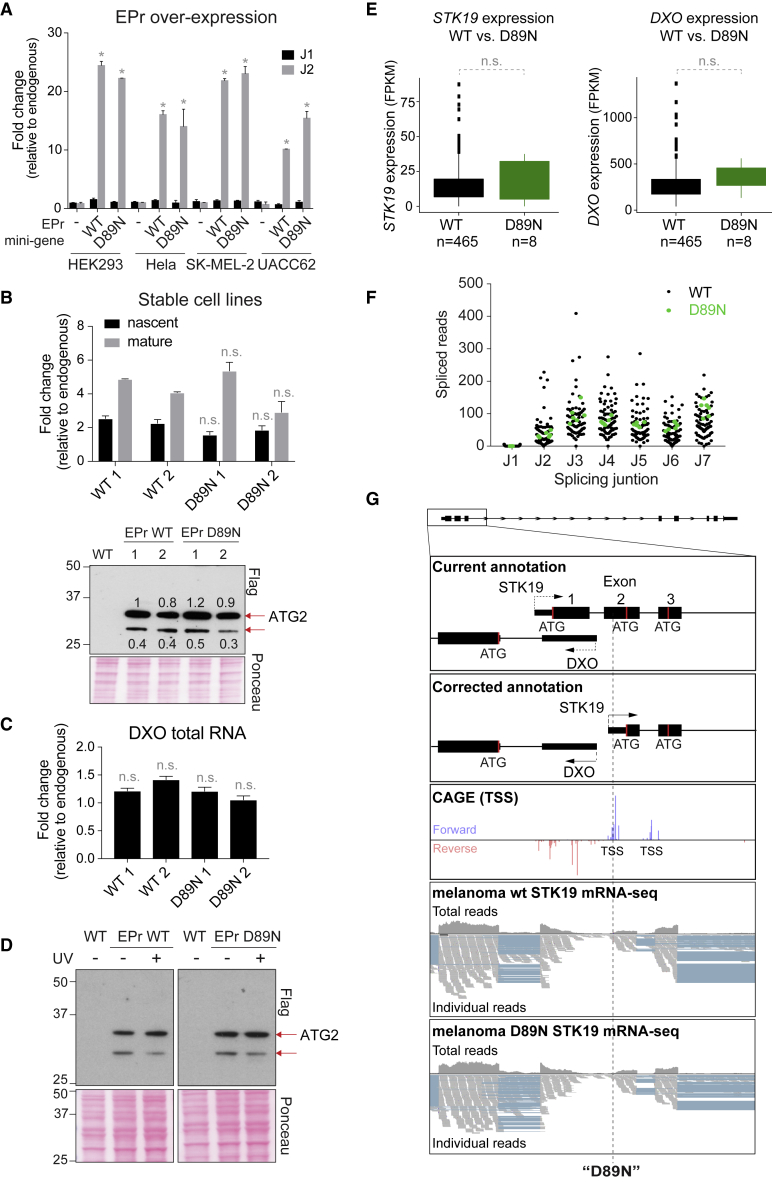
“D89N” Does Not Affect *STK19* Expression (A) qPCR of *STK19* expression after transient transfection of empty vector, WT or “D89N” EPr constructs (shown relative to endogenous control), comparing splice junction 1 (J1) and 2 (J2) as per the current annotation. Error bars represent ± SD. Statistically significant differences (p < 0.05, multiple t tests, Holm-Sidak correction) of three replicates compared to endogenous non-transfected control are indicated with asterisks. All other differences are not significant. (B) Top: qPCR of *STK19* expression in stable cell lines expressing a single copy of EPr WT or EPr “D89N.” Two cell lines of each construct are represented, showing relative expression of mature and nascent RNA compared to endogenous levels. Error bars represent ± SD. “n.s.” indicates non-significant differences compared to EPr WT (multiple comparison test, Holm-Sidak correction). Bottom: western blot of the same cell lines, using Flag antibody. Red arrows indicate the isoforms expressed. Bands at 34 and 29 kDa correspond to expression from the 2^nd^ and 3^rd^ ATG (or possibly a degradation product), respectively. Relative abundance of every band is shown. Ponceau-stained loading control is shown. (C) qPCR measuring *DXO* expression in cell lines stably expressing a single copy of EPr WT or EPr “D89N” relative to endogenous un-transfected control. Error bars represent ± SD. n.s indicate non-significant difference compared to EPr WT (Multiple t tests, Holm-Sidak correction). (D) Western blot of stable cell lines expressing EPr WT or EPr “D89N,” comparing STK19 expression before and 4 h after UV irradiation. Red arrows indicate the isoforms expressed. Bands at 34 and 29 kDa correspond to expression from the 2^nd^ and 3^rd^ ATG (or possibly a degradation product), respectively. (E) Left, *STK19* expression in melanoma patient samples with WT STK19 (n = 81) compared to those containing the “D89N” mutation (n = 6). Difference shown as non-significant (n.s.), Wilcoxon, p = 0.95. Right graph, same as left but for expression of *DXO*. Differences are not significant (n.s.), Wilcoxon, p = 0.078. (F) Splice read counts for every splice junction (J1 to J7) of mRNA-seq of patient samples with WT STK19 (black dots) and “D89N” STK19 (green dots). J1 counts in “D89N” samples is zero. (G) Diagram of *STK19* focusing on the 5′ region of the gene. From top to bottom, current annotation, corrected annotation, CAGE data, mRNA-seq data of a STK19 WT melanoma cell line followed by a melanoma cell line containing “D89N” mutation. The location of “D89N” is shown by a dashed line (see also Figure S4).

Although we show above that the *STK19* mutation annotated as “D89N” is not in the coding region of *STK19*, the mutation might conceivably affect *STK19* expression, either at the level of transcription or translation. To address this possibility, we introduced the “D89N” (C→T) mutation in the EPr mini-gene system (Figure 3A). As above, *STK19* gene expression was analyzed by RT qPCR after transient transfection, and splice junction 1 (J1) was used as a readout of isoform expression. Again, only background levels of J1 could be detected, both with mutant and WT *STK19* (Figure 4A), indicating that the mutation has no effect on start-site selection and isoform splicing. Next, we analyzed the possible changes “D89N” might cause to mRNA- and protein-expression. To do so, we took advantage of the Flp-In system (ThermoFisher) to generate HEK293 cell lines containing a single copy of the EPr transgene in a defined genomic location, to ensure that any expression changes were not due to differences in the number of gene copies or the location of integration between cell lines. Analysis by RT qPCR and western blot analysis of 2 WT clones and 2 “D89N” clones showed no evidence for changes in gene- or protein-expression upon introduction of the “D89N” mutation (Figure 4B, upper and lower panels, respectively). No changes in expression of the upstream *DXO* gene were observed either (Figure 4C). Moreover, because “D89N” appears to be a UV-induced mutation (see below), we also tested whether it affects the expression of *STK19* after UV irradiation. Again, western blot analysis showed little or no difference in *STK19* expression after UV-irradiation, neither from EPr WT nor from EPr “D89N” (Figure 4D).

**Figure S4:**
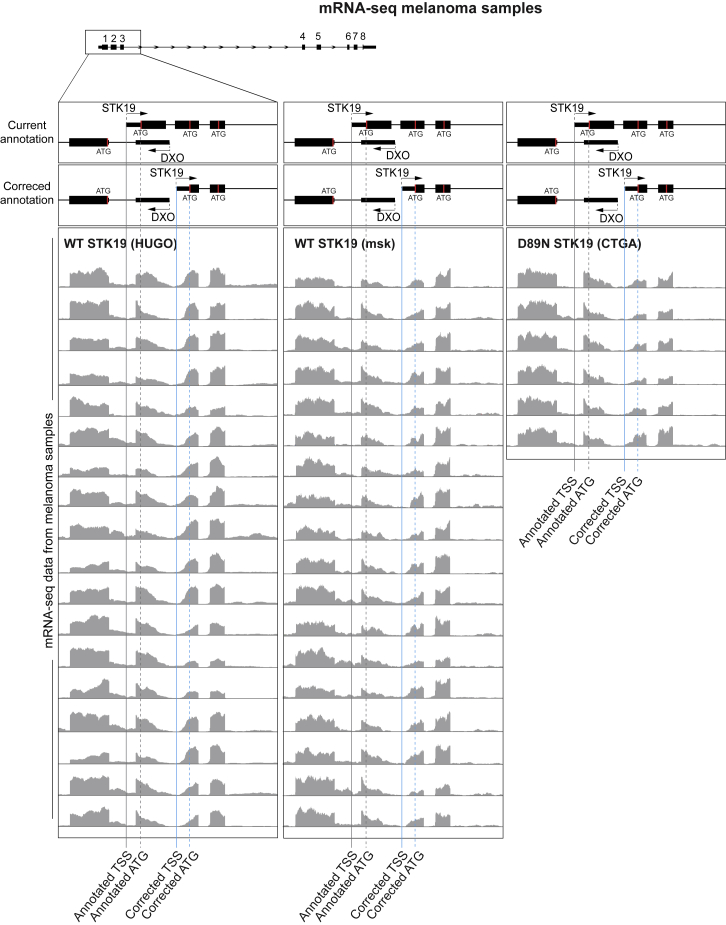
mRNA-seq Data from Melanoma Patient Samples to Compare wt STK19 and “D89N” STK19, Related to Figure 4 Genome browser view of *STK19*’s 5′ region. *STK19* 5′ and *DXO* 5′ are visible. Current and corrected annotations are shown aligned to mRNA-seq of melanoma patient data. Data from two different datasets are shown for WT STK19 (18 samples each), and all 6 samples of “D89N” found in the CTGA database. TSS position is marked with a line for currently annotated (black) and corrected annotation (blue), and start codon (ATG) is marked by a dashed line for currently annotated (black) and corrected annotation (blue).

Because the “D89N” mutation has been detected only in melanoma samples, we also analyzed melanoma RNA-seq data from patients to compare *STK19* expression levels in the presence or absence of “D89N.” Although the number of cancer samples is low (n = 8; the full available cohort of TCGA melanoma samples), statistical analysis showed no significant difference in gene expression in patient samples containing “D89N,” for either *STK19* or the upstream *DXO* gene (Figure 4E; p = 0.95 and p = 0.078, respectively, Mann Whitney U test). We also used these data to investigate the expression of the first splicing junction in patient samples. “D89N” mutation does not increase the read number at the first splice junction; indeed, no J1 reads were found in the “D89N” samples (Figure 4F). Visual analysis of “D89N” and WT raw sequencing reads also further confirms no notable difference in isoform- or gene expression levels (see Figure 4G for a representative sample, and Figure S4 for all samples).

Taken together, these data indicate that the presence of the “D89N” mutation in the promoter of *STK19* neither affects gene- nor protein-expression, and it therefore seems highly unlikely to be a melanoma driver mutation. Therefore, the data reported by Yin et al. (2019) on effects of this mutation on NRAS activation and melanoma progression may need to be reconsidered.

### Evidence That “D89N” Is a Common UV Signature Mutation

The data above indicate that the “D89N” mutation is not in the STK19 coding region, and this promoter mutation does not affect isoform-, gene- or protein-expression either. However, the nature of the “D89N” mutation remained unclear.

Interestingly, Fredriksson et al. (2017) recently showed that certain recurrent promoter mutations in melanoma are defined by a context-specific mutational signature. These mutations, located around the TSS of several genes, are caused by UV-irradiation and occur almost exclusively at cytosines flanked by the sequence signature TTCCG, and they do not have a functional role in gene expression. These mutations can be induced simply by UV-irradiating cells (Elliott et al., 2018, Fredriksson et al., 2017). Moreover, this specific mutation type is typically found in genes that remain actively transcribed after UV-irradiation, presumably as a consequence of transcription factors binding to this motif (Elliott et al., 2018, Fredriksson et al., 2017), which might prevent the association of the repair machinery with specific DNA sequences, as previously reported (Elliott et al., 2018, Perera et al., 2016, Sabarinathan et al., 2016).

**Figure 5:**
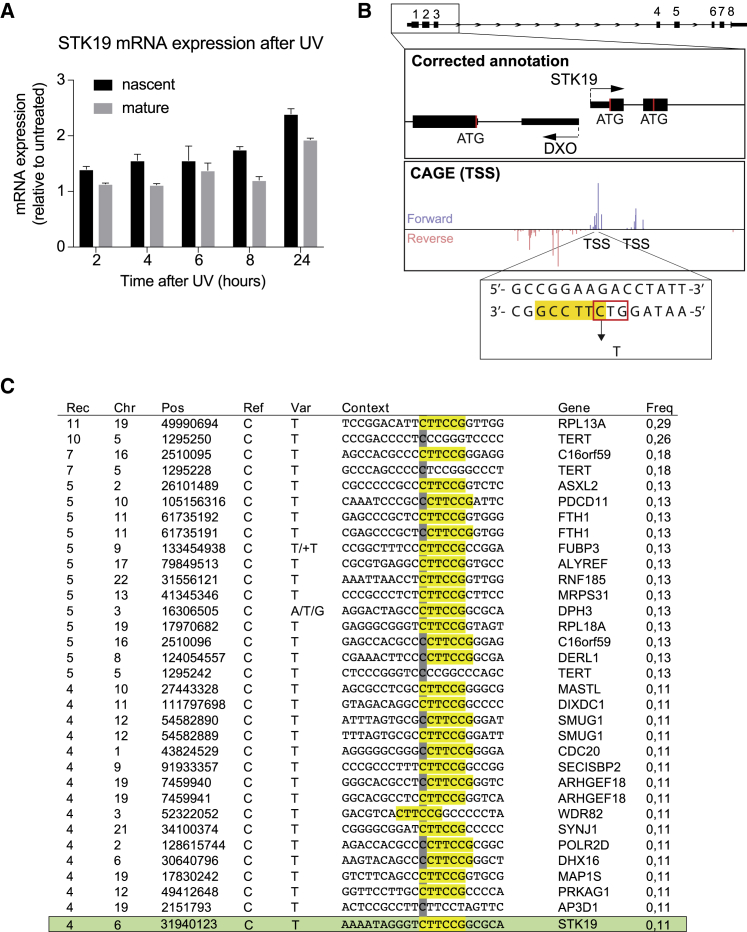
The “D89N” Mutation Is a Recurrent UV Signature (A) qPCR of *STK19* expression after UV irradiation relative to untreated and normalized to GAPDH. Nascent and mature RNA are shown. Error bars represent ± SD. Statistically significant differences (p < 0.05, multiple t tests, Holm-Sidak correction) indicate there is not downregulation of the gene in any of the time points analyzed. (B) Diagram of *STK19* gene 5′ region showing the corrected annotation aligned to CAGE (TTS) data and indicating the position of the “D89N” triplet mutation (red box). UV signature motif is underlined in yellow. (C) Table adapted from Fredriksson et al. (2017) showing all highly recurrent mutations within ± 500bp from TSS ordered by recurrence (number of mutated tumors). Based on the data used in Fredriksson et al. (2017), *STK19* was analyzed and added as the last row to show the similarities with sequences reported in their work. Rec, recurrence of each mutation. Chr, chromosome location. Pos, position. Var, variant base. Context, sequence context, showing pyrimidine-containing strand with respect to the central mutated base (gray). The motif CTTCCG is highlighted in yellow. Gene, closest gene to the mutation. Freq, frequency of the mutation. Note that the TERT promoter mutation is also listed, although not in the same sequence motif, and that mutations in the TERT promoter have recently been found in benign skin nevi, arguing against a role of such mutation in cancer progression (Colebatch et al., 2019).

Interestingly, the *STK19* “D89N” mutation fulfils all the criteria for being such a context-specific promoter mutation. The *STK19* gene continues to be expressed after UV irradiation (Figure 5A); the “D89N” mutation is a UV-signature mutation (a C→T transition); and it is located near the TSS (Figure 5B). Strikingly, the STK19 mutation actually occurs at a TTCCG motif and has a frequency of occurrence similar to the other mutations of the same kind described by Fredriksson et al. (2017) (Figure 5C).

Viewed in light of the experimental results above, these observations suggest that a mutation which previously resulted in *STK19* being classed as a cancer driver is merely a UV-signature promoter mutation that has no functional consequence.

### STK19 Is a Nuclear Protein; Has Little or No Effect on MEK-AKT Signaling; and Does Not Appear to Be a Kinase

**Figure 6:**
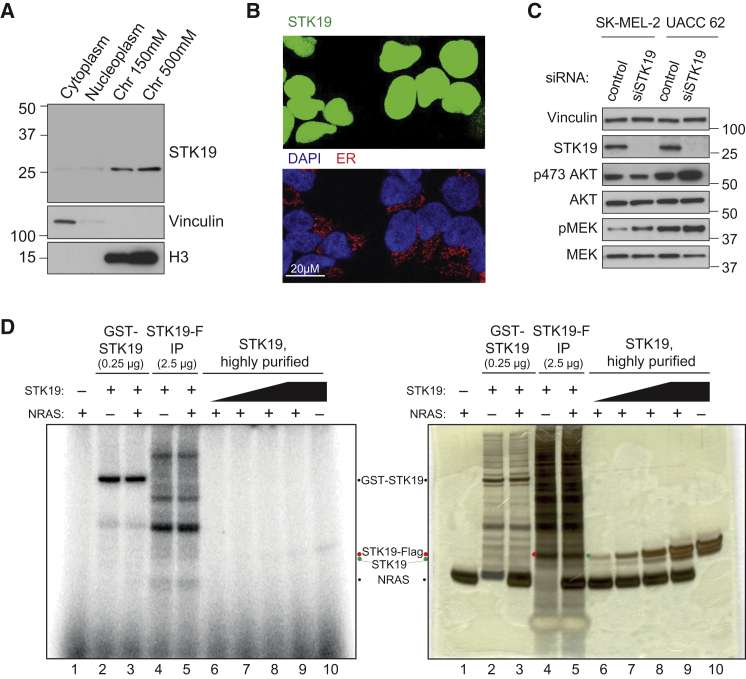
Evidence That STK19 Is Chromatin-Associated and That It Binds DNA (A) STK19 western blot analysis after sub-cellular fractionation. “Chromatin 150 mM” and “Chromatin 500 mM” contain proteins extracted from the chromatin using 150 mM or 500 mM NaCl, respectively. Vinculin and histone H3 (H3) are used as markers for predominantly cytoplasmic and chromatin localization, respectively. (B) Detection of STK19-flag-GFP (green) by microscopy. Image is overexposed to enable detection of any possible GFP specific signal outside the nucleus. DAPI-stained nuclei (blue) and ER calnexin (red) are shown as controls. (C) Western blot analysis of AKT and MEK phosphorylation (p473 AKT and p217/221 MEK) before and after STK19 knockdown and their respective unphosphorylated controls. Vinculin is used as control. Please note that the blot-strip depicting STK19 and the loading control, vinculin, are repeated from Figure 2D, as these came from the same experiment. (D) STK19 kinase reactions, utilizing the conditions used by Yin et al., 2019, with highly purified NRAS protein as the substrate. STK19-FLAG (STK19-F) was isolated from cells using the procedures described by Yin et al., while GST-STK19 was purchased from SignalChem. The autoradiograph on the left was generated by exposure of the silver-stained gel on the right. The migration of relevant proteins is indicated between the images. Note the many proteins co-immunoprecipitating with STK19 using Yin et al.’s conditions (lanes 4–5).

NRAS activates cytoplasmic signaling pathways by recruiting effectors to the plasma membrane (Prior and Hancock, 2012). However, we find that STK19 is nuclear and very tightly chromatin-associated (Figure 6A and B). Indeed, even at high levels of overexpression, we failed to observe GFP-tagged STK19 in the cytoplasm or at membranes (Figure 6B). This is at odds with the proposed role for STK19 in phosphorylation of oncogenic NRAS at the plasma membrane (Yin et al., 2019), but agrees with previous data on the presence of a bi-partite nuclear localization motif in the protein sequence of STK19 and its sub-cellular localization (Gomez-Escobar et al., 1998).

As described above, the majority of the data shown by Yin et al. (2019) were obtained using the 41 kDa D89N STK19 isoform, which we argue is a physiologically irrelevant protein isoform. We investigated the possibility that the 29 kDa STK19 isoform might have an effect on NRAS pathway activation, in spite of its predominantly nuclear localization. For this purpose, we repeated an experiment performed by Yin et al., which investigated the effect of STK19 knockdown on NRAS signaling to MEK and AKT kinases in cell lines that either carry WT NRAS or the NRAS Q61R mutation. In their paper (Figures S1B, S1E and S1F), Yin et al. (2019) showed that STK19 knockdown in cells with NRAS Q61R mutation dramatically decreased MEK and AKT phosphorylation. In our repeat of this straight-forward knockdown/Western blot experiment, we were unable to detect any notable change in the phosphorylation events signifying NRAS activation, using the same cell lines as Yin et al. (2019), in spite of efficacious STK19 depletion (Figure 6C).

As indicated by its name, STK19 (serine-threonine kinase 19) is annotated as a protein kinase, and Yin et al. (2019) developed an STK19-targeted small molecule kinase inhibitor and provided evidence that this inhibitor blocks oncogenic, NRAS-driven melanocyte malignant transformation and melanoma growth. Unfortunately, the kinase assays in Yin et al. (2019) were performed only with crude STK19-precipitates from cell extracts, or with semi-purified 41 kDa protein, at least partly from a commercial source (which, according to the manufacturer (Signal Chem), is only “>70% pure” (see Figure 6D, lanes 2–3), but never with highly purified, recombinant protein, or across chromatography fractions to provide evidence that the activity is indeed due to STK19 rather than a co-precipitated/contaminating protein. In all their experiments, it was the 41 kDa STK19 protein that was tested. This “unnatural” STK19 version contains a (conceivably unfolded) N-terminal domain, which is not normally part of the protein. Indeed, in our hands, the 41 kDa protein isoform shows low solubility and is unstable. By contrast, 29 kDa STK19 protein can be purified to virtual homogeneity and is stable and soluble. We tested whether the 29 kDa STK19 protein might have kinase activity. We also tested the commercially available GST-STK19 successfully used by Yin et al. (Signal Chem; 41 kDa STK19), as well as STK19-FLAG (29 kDa version) from human cells extracts, isolated by immuno-precipitation employing Yin et al.’s conditions. The kinase assays were performed as described by Yin et al. as well, with highly purified NRAS as the substrate (Figure 6D). Although background, radioactively labeled bands of uncertain origin were detected when using the impure STK19 fractions (lanes 2–5), no NRAS-specific signal was detected in any of the reactions (Compare reactions containing NRAS to controls without it (lane 2 versus lane 3; lane 4 versus lane 5; and lanes 6–9 versus lane 10, respectively)). These results suggest that STK19 is not an NRAS kinase.

Together, these data again indicate that the conclusions made by Yin et al. (2019) need to be reconsidered.

## Discussion

In this report, we provide evidence that *STK19* encodes a 29 kDa protein. This conclusion is based on multiple independent lines of evidence. First, STK19 amino acid conservation is strictly limited to the region encoding the 29 kDa protein; the N-terminal region of the presently annotated 41 kDa STK19 protein is absent in other metazoans. Second, genomic analyses (CAGE, TT-Seq, RNA-Seq, qPCR and deep-sequencing analysis of splice junctions) all indicate that the *STK1*9 TSS is located downstream of the junction of presently annotated exon 1 and 2. Third, mRNA analysis and protein analysis by mass spectrometry and western blotting of cells expressing the endogenous *STK19* gene or additionally containing a construct mimicking the exon structure at the beginning of the gene, confirm that only a 29 kDa STK19 protein is expressed, in a variety of cell lines. These conclusions hold true both in WT cells and in cells expressing the *STK19* “D89N” mutation. Importantly, we show that the “D89N” mutation is a UV-signature mutation (a C→T transition) located near the *STK19* TSS, which has no effect on neither transcription levels, mRNA splicing, nor translation. Together, these data indicate that the 41 kDa STK19 isoform is not expressed, and thus argue that experiments performed with the D89N “cancer driver” are physiologically irrelevant.

**Figure S5:**
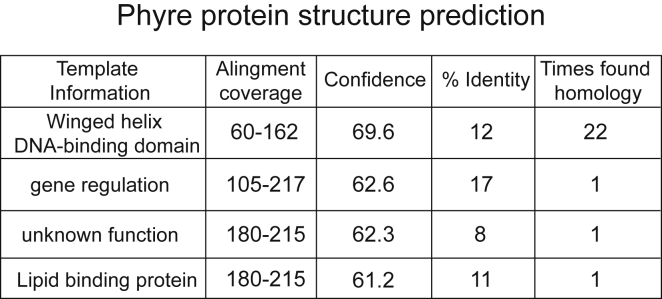
Table of the Phyre2 Prediction Results, Related to Figure 6 “Times found homology” corresponds to the times that Phyre finds that same prediction out of 100 results.

We also failed to find evidence that STK19 is an NRAS-directed protein kinase. It is obviously difficult to make strong conclusions based on negative results, but our repeated inability to detect kinase activity with STK19 *in vitro* prompted us to further investigate the basis for the idea that it is a protein kinase. This possibility was first suggested ∼20 years ago based primarily on similarity between STK19 (then called G11 or RP1) and the tyrosine kinase-transforming protein (TKFB) from Fujinami virus (Gomez-Escobar et al., 1998, Sargent et al., 1994). However, much has happened in the area of protein homology-modeling over recent decades, and by today’s standards this similarity simply is not significant. Indeed, we have performed numerous comparisons with STK19 using current software and have failed to uncover any homology to Fujinami virus TKFB, or any other kinase. Importantly, the STK19 homology previously uncovered was actually not even with the kinase domain of TKFB, i.e., amino acids 611–865, but instead with amino acids 191–340 of that protein (Sargent et al., 1994). Indeed, despite the high structural conservation of the catalytic domain of kinases (Knight et al., 2007, Taylor and Kornev, 2011), the protein sequence of STK19 cannot be convincingly aligned to any known protein kinase with current structural prediction tools such as Phyre (Kelley et al., 2015). Tellingly, a comprehensive list of all known (∼500) human kinases has been compiled (https://www.uniprot.org/docs/pkinfam), and STK19 is not among them. Instead, a structural model for STK19 can be generated based on sequence homology to winged-helix domains from DNA binding proteins (Figure S5). In agreement with this prediction, the purified 29 kDa protein indeed binds DNA (M.R.-M. and M.N.G., unpublished data). The mechanistic relevance of this result is presently being investigated, but we note that this finding is consistent with our previous data indicating that STK19 is recruited to DNA regions with UV-induced lesions (Boeing et al., 2016) and with its strong association with chromatin (Figure 6A).

In this connection, we also note that the “active site mutation” used by Yin et al. (2019) to negate STK19 kinase activity in their overexpression experiments *in vivo* is a lysine_317_ to proline change (in the annotated, 41 kDa STK19 isoform; K_207_ in the new, corrected annotation (29 kDa)). In kinases, a lysine in the catalytic site is found in the context of a highly conserved AxK kinase signature motif, which coordinates the ATP phosphates (Taylor and Kornev, 2011). By contrast, the STK19 K_317_ residue is found in the context of KAK_317_, making it highly unlikely to be part of a kinase catalytic site. A lysine→proline change will also potentially dramatically change the folding of the protein domain in question and might affect the STK19 interactome as well. Finally, we note that the NRAS phosphorylation site mapped by Yin et al. as being the target of STK19 kinase activity *in vitro*, NRAS serine 89, has not previously been detected by protein phosphorylation site mapping and is thus not listed among the NRAS phosphorylation sites in Phosphosite Plus, for example, questioning the physiological relevance of this *in vitro* phosphorylation event. Taken together, although the observations described above obviously cannot be taken as final proof that STK19 is not a protein kinase, they definitely suggest that this is unlikely and that this key issue needs to be investigated much more thoroughly before STK19 inhibitors are further developed.

In conclusion, the current STK19 annotation is incorrect. The only *STK19* gene product is a 29 kDa protein, and no convincing evidence to support the existence of a 41 kDa isoform was uncovered, neither in normal nor melanoma cells. Any data based on its exogenous expression is therefore arguably physiologically irrelevant. Moreover, the incorrect *STK19* annotation has led to it being wrongly identified as a melanoma driver. This underscores the importance of careful, manual curation of any newly identified cancer driver gene, as well as studies on healthy tissue to rule out any events unrelated to cancer when calling new drivers. Taken together, the multiple datasets and observation described herein challenge the conclusions recently published by Yin et al. (2019) in *Cell*.

## STAR★Methods

### Key Resources Table

**Table:** 

REAGENT or RESOURCE	SOURCE	IDENTIFIER
**Antibodies**		

Mouse monoclonal to Flag	Sigma-Aldrich	Cat#F3165; RRID: AB_259529
Mouse monoclonal to Vinculin	Sigma-Aldrich	Cat#V9131; RRID: AB_477629
Rabbit polyclonal to STK19	Novus Biologicals	Cat#NBP2-33955
Rabbit polyclonal to Histone H3	Abcam	Cat#ab1791; RRID: AB_302613
Anti-mouse HRP	Santa Cruz	Cat#sc-516102; RRID: AB_2687626
Anti-rabbit HRP	Jackson ImmunoResearch	Cat#711-035-152; RRID: AB_10015282
Anti-calnexin alexa 647	abcam	Cat#ab202572
Rabbit polyclonal to AKT	Cell signaling Technology	Cat#9272; RRID: AB_329827
Rabbit polyclonal to phosphor-AKT (ser473)	Cell signaling Technology	Cat#9271; RRID: AB_329825
Rabbit polyclonal to MEK1/2	Cell signaling Technology	Cat#9122; RRID: AB_823567
Rabbit polyclonal to phosphor-MEK1/2 (ser217/221)	Cell signaling Technology	Cat#9121; RRID: AB_331649

**Bacterial and Virus Strains**		

NEB 5-alpha Competent *E. coli*	NEB	Cat#C2988J
Rosetta 2(DE3) pLacI Competent Cells	Novagen	Cat#71404
Biological Samples		

**Chemicals, Peptides, and Recombinant Proteins**		

Doxycycline	Clontech	Cat#8634-1
Blasticidin	TOKU-E	Cat#B007
Hygromycin B	TOKU-E	Cat#H011
N-Ethylmaleimide (NEM)	Sigma-Aldrich	Cat#E3876
STK19 purified protein	This study	N/A
ULP-1	Kind gift from Peter Cherepanov	N/A
3xFLAG peptide	Peptide Chemistry, The Francis Crick Institute	N/A

**Critical Commercial Assays**		

RNeasy kit	QIAGEN	Cat#74104
RNase-Free DNase Set	QIAGEN	Cat#79254
Taqman Reverse Transcriptase Reagents	Thermo Fisher Scientific	Cat#N8080234

**Experimental Models: Cell Lines**		

Flp-In T-Rex HEK293 cells	Thermo Fisher Scientific	Cat#R78007
Flp-In T-Rex HEK293 EPr-STK19-2x3 flag cells	This study	N/A
Flp-In T-Rex HEK293 STK19-flag-GFP cells	This study	N/A
Flp-In T-Rex HEK293 mis-annotated STK19-flag cells	This study	N/A
Flp-In T-Rex HeLa cells	Kind gift from Stephen Taylor	N/A
UACC6 2 cell line	NCI-60 collection	N/A
SK-MEL-2 cell line	NCI-60 collection	N/A

**Oligonucleotides**		

siGENOME Human STK19 (8859) siRNA - SMARTpool	Dharmacon	Cat#M-005378-02
siGENOME Non-Targeting siRNA Pool #2	Dharmacon	Cat#D-001206-14
Full list in Table S2		

**Recombinant DNA**		

pFRT/TO	Kind gift from Markus Landthaler	N/A
EPr_STK19_2x3flag	This study	N/A
pOG44	Thermo Fisher Scientific	Cat#V600520
pSpCas9(BB)-2A-GFP	(Ran et al., 2013)	Addgene Cat#48138
pET28-His-SUMO	Kind gift from Peter Cherepanov	N/A

**Software and Algorithms**		

SGSeq v1.16.2	Goldstein et al., 2016	http://www.bioconductor.org/packages/release/bioc/html/SGSeq.html

**Other**		

SuperSignal West Pico PLUS ECl reagent	Thermo Fisher Scientific	Cat#34577
SuperSignal West Dura ECl reagent	Thermo Fisher Scientific	Cat#34075
Radiance Plus Femtogram HRP substrate	Azure Biosystems	Cat#AC2103
High glucose DMEM	Thermo Fisher Scientific	Cat#11965118
RPMI-1640 with L-glutamine	Sigma-Aldrich	Cat#R8758-500ML
Poly-lysine	Sigma-Aldrich	Cat#P7280
4–15% TGX gels (18wells/26/wells)	BioRad	Cat#56711084/5
Complete EDTA-free protease inhibitor cocktail	Sigma-Aldrich	Cat#05056489001
PhosSTOP	Sigma-Aldrich	Cat#04906837001
Nitrocellulose membrane	GE Healthcare Life Sciences	Cat#10600002
Benzonase	MerckMillipore	Cat#70746-4
Lipofectamine 2000	Thermo Fisher Scientific	Cat#11668019
Lipofectamine 3000 Reagent	Thermo Fisher Scientific	Cat#L3000001
Lipofectamine RNAiMAX Transfection Reagent	Thermo Fisher Scientific	Cat#13778150
In-Fusion HD cloning Kit	Takara Clontech	Cat#639649
iTaq Universal SYBR Green Supermix	BioRad	Cat#172-5124
Q5 Site-Directed Mutagenesis Ki	NEB	Cat#E0554S
Ni-NTA agarose	QIAGEN	Cat#30230
HiTrap SP HP	GE healthcare	Cat#17115201
Superdex 200	GE healthcare	Cat#17517501
ANTI-FLAG M2 Affinity Gel	Sigma-Aldrich	Cat#A2220
VECTASHIELD Antifade Mounting Medium-DAPI	Vector Laboratories	Cat#H-1200

### Resource Availability

#### Lead contact

Further information and requests for resources and reagents such as plasmids should be directed to and will be fulfilled by the Lead Contact, Jesper Q. Svejstrup (jesper.svejstrup@crick.ac.uk).

#### Materials availability

All materials and reagents generated in this paper are available upon request to the lead contact stated above.

#### Data and code availability

This study did not generate new data. Public data used is specified under every method in the quantification and statistical analysis section.

### Experimental Model and Subject Details

#### Cell lines and culture conditions

SK-MEL-2 and UACC62 cells lines (NCI-60 collection) were maintained in RPMI-1640 with L-glutamine (Sigma R8758-500ML) supplemented with 10% v/v FBS, 100 U/mL penicillin, 100 μg/mL streptomycin. For simplicity purpose we refer to Flp-In T-REx HEK293 cells as HEK293 and to Flp-In T-REx HeLa cells as HeLa in the results and figures sections. Flp-In T-REx HEK293 cells (Thermo Fisher Scientific, R78007, human embryonic kidney epithelial, female origin) and Flp-In T-REx HeLa cells (these were a kind gift from Stephen S. Taylor, (Tighe et al., 2004)) were cultured in high glucose DMEM (Thermo Fisher Scientific, 11965118) supplemented with 10% v/v FBS, 100 U/mL penicillin, 100 μg/mL streptomycin and 100 μg/mL zeocin and 15 μg/mL blasticidin were cultured at 37°C with 5% CO2 and routinely passaged 2–3 times a week. All cell lines were confirmed to be mycoplasma-free.

### Method Details

#### Plasmid construction

The EPr mini-gene construct was generated by gene synthesis (Genscript). After removal of CMV promoter (NruI/XhoI) from pFRT/TO vector, EPr was cloned into pFRT/TO without CMV promoter by In-Fusion system (Takara 639649) following manufacturer instructions. C-terminal flag-tagged misannotated STK19 was generated by Genscript, and cloned into pFRT/TO (using EcoRV and xhoI sites). STK19 was generated by deleting the first 110 aminoacids of the misannotated STK19 using Q5 site directed mutagenesis (NEB, E0554S) and addition of GFP was done using In-Fusion system (Takara 639649). STK19 (29kDa) codon optimized for bacteria was generated by Genscript and cloned into pET28-His-SUMO using BamHI and EcoRI sites. Primers used for cloning and final DNA sequences are listed in Table S1.

#### Protein alignment and protein structure prediction

STK19 protein sequences from *Homo sapiens* (GenBank: NP_004188), *Mus musculus* (GenBank: NP_062315), Danio rerio (GenBank: NP_001108564) and *Xenopus laevis* (GenBank: NP_001088743) were aligned using MuscleWS (MUSCLE v3.8.31) (Edgar, 2004), visualized using Jalview 2 and colored by protein identity. Structure prediction for STK19 (UniProt P49842-4) was done using Phyre2 (Kelley et al., 2015) intensive modeling mode.

#### Generation of stable cell lines

Flp-In T-REx HEK293 cell lines expressing doxycycline inducible STK19-flag-GFP, misannotated STK19-flag or EPr-wt-STK19-2x3flag, EPr-D89N-STK19-2x3flag mini-genes and were generated as described previously (Gregersen et al., 2019). Briefly, Flp-In T-REx HEK293 cell lines maintained in 100 μg/mL zeocin and 15 μg/mL blasticidin prior to transfection, were co-transfected with a 9:1 ratio of pOG44 Flp-recombinase expression vector (Thermo Fisher Scientific, V600520) and pFRT/TO/STK19-flag-GFP, pFRT/TO/mis-annotated_STK19-flag or EPr-STK19-3x2flag hygromycin resistant constructs using Lipofectamine 2000 (Thermo Fisher Scientific, 11668019) according to the manufacturer’s instructions. 24 h after transfection, cells were seeded as single cells and after another 24 h the cell culture media was supplemented with 100 μg/mL hygromycin (H011, TOKU-E) and 15 μg/mL blasticidin (B007, TOKU-E). Expression of GFP-tagged proteins was induced overnight by the addition of doxycycline (Clontech, 8634-1, 1 μg/mL final concentration) and all clones were verified by western blotting using antibodies against GFP, flag and/or STK19. CRISPR-Cas9-nuclease-mediated genome editing was performed in Flp-In T-REx HEK293 and Flp-In T-Rex HeLa cell lines. The oligonucleotide encoding the gRNA for targeting the coding region of STK19 is described in Table S1. The gRNA was annealed and ligated into pSpCas9(BB)-2A-GFP ((Ran et al., 2013) Addgene, PX458), and plasmids were sequenced after cloning and transformation. To generate knockouts, cells were transfected with pSpCas9(BB)-2A-GFP plasmids expressing the gRNA, EGFP and Cas9 using Lipofectamine 2000 (Thermo Fisher Scientific, 11668019) according to the manufacturer’s instructions. 48 h after transfection, high GFP positive cells were sorted clonally by fluorescence activated cell sorting (FACS) into 96-well plates and cultivated until colonies were obtained. Genomic PCRs around the edited site were sequenced and analyzed using the Web tool “TIDE” (https://tide.deskgen.com). Cells containing Indels were expanded from the master plate for further analysis by western blot. The expression of the upstream gene DXO was analyzed by RT qPCR to confirm that the mutation in STK19 is not affecting the expression of the upstream gene.

#### UV irradiation conditions

For UV irradiation experiments, cells were irradiated using an in-house built conveyor belt with 10 or 15 J /m^2^ UVC for Flp-In T-REx HeLa cells and Flp-In T-REx HEK293 cells respectively and analyzed 4 h later or indicated time points.

#### siRNA and transient transfections

Cells were transfected with STK19 siRNA (siGENOME SMARTpool, Dharmacon M-005378-02) or non-targeting control (siGENOME Non-Targeting siRNA Pool #2, Dharmacon D-001206-14) using RNAiMAX transfection reagent (Thermo Fisher Scientific 13778030) following manufacturer instruction. Briefly, cells were seeded at 40% confluency in 6 well plates and transfected with 50nmol (FlpIn T-Rex HEK293) or 15nmol (FlpIn T-Rex HeLa, SK-MEL-2 and UACC_62) and knock down efficiency assayed 72 h after transfection. For transfection with EPr mini-gene constructs and empty vector controls, cells at 50% confluency were seeded in 6 well plates and transfected using Lipofectamine 2000 (ThermoFisher Scientific 11668027) following manufacturer instructions, and analyzed 24 h later.

#### Quantitative PCR (qPCR)

Total RNA was extracted using the RNeasy kit (QIAGEN, 74104) for nascent and mature RNA, following the instructions of the manufacturer including an on-column DNase treatment (QIAGEN, 79254). Reverse transcription was performed using TaqMan Reverse Transcription Reagents (Thermo Fisher Scientific, N8080234). For detection of nascent transcripts, random hexamers were used for the reverse transcription step; for mature mRNA, oligo dT primers were used. cDNA was amplified using iTaq Universal SYBR Green Supermix (BioRad, 172-5124) with 30 cycles of 15 s denaturation at 94°C, 15 s annealing at 58°C, and 20 s extensions at 72°C. Primers amplifying mature *GAPDH* were used as normalization control. Unless differently stated, ΔCT values were calculated relative to GAPDH before normalizing to the expression level in control sample and experiments were done in triplicate. Error bars show SD. Primers to amplify nascent RNA were spanning genomic exon-intron regions, and for mature RNA were spanning exon-exon junctions. Primer sequences are listed in Table S1.

#### Whole cell extract preparation, cell fractionation and western blotting

For whole cell extracts, cells pellets were lysed in NP-40 lysis buffer (50 mM Tris-HCl pH 7.5, 500 mM NaCl, 2 mM EDTA, 0.5% (v/v) NP-40, 0.5 mM DTT, PhosSTOP (Sigma-Aldrich, 04906837001) and Protease Inhibitor Cocktail (Sigma-Aldrich, 05056489001). Cell fractionation was performed as previously described (Gregersen et al., 2019). 30–100 μg protein/lane was separated on 4%–15% TGX gels (BioRad, 56711084/5) and transferred to nitrocellulose membranes (GE Healthcare Life Sciences, 10600002). Membranes were blocked in 5% (w/v) skimmed milk in PBS-T (PBS, 0.1% (v/v) Tween20) for 1 h at room temperature and incubated with primary antibody (in 5% (w/v) skimmed milk in PBS-T) overnight at 4°C. Primary antibodies are listed in key resources table. Antibody against vinculin were used to control loading. Membranes were washed several times in PBS-T, incubated with HRP-conjugated secondary antibody in 5% (w/v) skimmed milk in PBS-T and visualized using SuperSignal West Pico PLUS (for Vinculin and H3), Dura (for flag) Chemiluminescent Substrate ECL reagent (Thermo Fisher Scientific, 34577 or 34075) or Radiance Plus Femtogram HRP substrate (for endogenous STK19) (Azure Biosystems, AC2103). When stated in the text, western blot were performed using the methods provided by Yin et al.

#### Immunoprecipitations of misannotated STK19 for mass spectrometry analysis

Flp-In T-REx HEK293 cells stably expressing doxycycline (Dox)-inducible misannotated STK19-flag were induced overnight by the addition of Dox (1000,100,10 or 1 ng/mL final concentration). Cells were harvested by scraping in ice-cold PBS, washed once in cold PBS and pelleted by centrifugation at 1,500 rpm for 5 min at 4°C. Cells were then fractionated as previously described and all nuclear fractions were pooled to enrich in STK19. Phosphatase inhibitors (PhosSTOP, Sigma-Aldrich, 04906837001) and Protease Inhibitor Cocktail (Sigma-Aldrich, 05056489001) were added fresh to all buffers. Flag immunoprecipitation was done by incubating nuclear fractions with ANTI-FLAG M2 Affinity Gel (Sigma-Aldrich, A2220) at 4°C for 3 h. Beads were washed 5 times in IP wash buffer (150 mM NaCl, 20 mM Tris-HCl pH 7.5, 1.5 mM MgCl2, 3mM EDTA, 10% (v/v) glycerol, 0.1% (v/v) NP-40, phosphatase inhibitors (PhosSTOP, Sigma-Aldrich, 04906837001) and protease inhibitor cocktail (Sigma-Aldrich, 05056489001)) with the last wash being on a spin column (Thermo Fisher Scientific, 69705). Immunoprecipitates were eluted using 1 mg/mL 3xFLAG peptide dissolved in IP wash buffer by incubation for 1 h at 4°C. FLAG elutions were fractionated on SDS-PAGE, analyzed by western blot, or stained using the SilverQuest Silver Staining Kit (Thermo Fisher Scientific, LC6070) to confirm immunoprecipitation of full-length misannotated STK19. Samples were then sent for mass-spectrometry analysis of detected peptides.

#### Microscopy

STK19-GFP expressing cells were seeded onto poly-lysine (Sigma-Aldrich, P7280) coated coverslips in Doxycycline-containing media (1 μg/mL). Cells were fixed using 4% (v/v) formaldehyde in PBS for 15 min, blocked in PBS-T-BSA (PBS, 0.1% (v/v) Tween20, BSA 1%) for 1 h at RT, incubated with anti-Calnexin Alexa Fluor-647 conjugated antibody (key resources table) for 1 h in PBS-T-BSA, washed 3 times in PBS and mounted onto slides using VECTASHIELD Antifade Mounting Medium containing DAPI (Vector Laboratories, H-1200) and visualized using an upright 780 confocal Zeiss microscope. FIJI was used to analyze the images.

#### Generation of STK19 (29 kDa) baculovirus and protein expression

The coding sequence of STK19 (110-368) with a 6xHis followed by a Twin Strep(II) tag (WSHPQFEKGGGSGGGSGGSAWSHPQFEK) was inserted into the pFL vector (Fitzgerald et al., 2006) by Genescript. Sequence is available in Table S1. A HRV 3C protease cleavage site is present to proteolytically remove the tag. A baculovirus stock was generated by transposition of pFL_Stk19_3C_Strep into DH10Bac cells (ThermoFisher). Bacmid DNA was prepared as previously described (Fitzgerald et al., 2006) and used to transfect Sf21 cells maintained in SF900-III medium (ThermoFisher) at 27°C with 120 rpm shaking. The baculovirus stock was passaged to a titer of approximately 10^8^ pfu/mL and used to infect 1 L of Sf21 cells at 1 × 10^6^ cells/mL with an MOI of 2 for 72 h. The infected cells were harvested by centrifugation at 1000 × g for 10 min and the pellet was flash-frozen and stored at −80°C.

#### Purification of STK19

The infected cells were re-suspended in 1/20th the original culture volume in a buffer consisting of 50 mM HEPES, pH 7.4, 150 mM NaCl, 1 mM DTT. An EDTA-free protease inhibitor tablet (Roche) was added to each 50 mL of buffer. The cell suspension was briefly sonicated on ice, using a Branson 550 sonicator using 5 s pulse on/10 s pulse off cycles for a total of 1 min. Subsequently, 10kU of BaseMuncher (Expedeon) was added and incubated for 1 h at 4°C with gentle agitation. Thereafter, NaCl was added to a final concentration of 1 M to dissociate bound DNA from STK19. Additionally, EDTA was added to a final concentration of 1 mM and the lysate incubated for 2 h at 4°C. Insoluble material was removed by centrifugation at 80K x g for 30 min. The cleared lysate was filtered through a 0.45 μm filter and applied to a 1 mL StrepTrap column (GE Healthcare) using a running buffer consisting of 50 mM HEPES, pH 7.4, 150 mM NaCl, 1 mM DTT and 1 mM EDTA. Upon washing, STK19 was eluted with a running buffer containing 10 mM desthiobiotin (Sigma – D1411). The Twin-Strep and 6xHis tag was removed by digestion with recombinant GST-3C protease overnight at 4°C. Subsequently, the protein was diluted in 10 mM NaH_2_PO_4_, pH 7.0, 1 mM DTT and 1 mM EDTA before being applied to a 1 mL Heparin column (GE Healthcare) using in 10 mM NaH_2_PO_4_, pH 7.0, 20 mM NaCl, 1 mM DTT and 1 mM EDTA as running buffer and eluting with a gradient from 20–1000 mM NaCl. STK19 eluted at 50 mS/cm. For further purity and to separate full-length STK19 from an N-terminally proteolytically clipped variant, STK19 was subjected to cation chromatography using a MonoS (5/50 GL) column (GE Healthcare). A buffer consisting of 50 mM HEPES, pH 7.4, 50 mM NaCl, 1 mM EDTA and 1 mM DTT was used as running buffer and again a NaCl gradient was applied from 50–1000 mM NaCl. Intact STK19 eluted at 55 mS/cm and was concentrated before being injected onto a Superdex75(GE Healthcare) column in a running buffer of 50 mM HEPES, pH 7.4, 250 mM NaCl, 1 mM DTT and 1 mM EDTA. STK19 eluted as a monomer, (which was also confirmed by SEC-MALLS), and was concentrated to a final concentration of 1 mg/mL with a A_260_/A_280_ ratio of 0.6.

#### NRAS cloning, expression and purification

Human NRAS full-length cDNA was cloned into pGEX-6P-1, followed by transformation into BL2-CodonPus (DE3)-RIL (Agilent Technologies 230245). A single colony was picked and inoculated in LB (Lysogeny Broth) media overnight at 37°C. The next day, the culture was diluted 1:100 in LB media and incubated at 37°C until OD_600_ reached 0.6–0.7, then induced with 0.5mM IPTG (Isopropyl β- d-1-thiogalactopyranoside) and incubated overnight at 18°C. The next day, the culture was harvested, and resuspended in lysis buffer (50 mM Tris pH 7.5, 250 mM NaCl, 2 mM MgCl2, 0.5 mM TCEP, 0.5 mg/mL lysozyme, protease inhibitors cocktail (Sigma). The lysate was then sonicated, centrifuged at 18,000 rpm for 45min and the supernatant was incubated with glutathione agarose resin (GE Healthcare) for 45 min at 4°C. The resin was then washed with washing buffer (50 mM Tris pH 7.5, 250 mM NaCl, 2 mM MgCl2, 0.5 mM TCEP), and the NRAS was cleaved of the GST-tag and beads using HRV 3C protease (produced in house) overnight incubation at 4°C. NRAS was then loaded with 20x molar excess of either GppNHP (Sigma G0635-5MG) or GDP (Sigma G7252) in the presence of EDTA (5 mM final concentration) and incubation for 30min at 30°C, followed by the addition of MgCl2 (10 mM) on ice. Loaded NRAS was then followed by a second step purification gel filtration using a S75 16/60 column in buffer (25 mM Tris pH 7.5, 100 mM NaCl, 2 mM MgCl2, 0.5 mM TCEP). The protein fractions were then concentrated, aliquoted, snap frozen in liquid nitrogen and stored at −80°C.

#### Immunoprecipitation of STK19-Flag

A plasmid expressing STK19-flag was transfected into HEK293 cells, and STK19 was immunoprecipitated following the method of Yin et al., 2019. Briefly, cell pellets were collected from 3 × 100 mm dishes and lysed by gentle agitation for 1 h at 4°C with 10 mL ice-cold NETN buffer (20 mM Tris-HCl pH 8.0, 100 mM NaCl, 1 mM EDTA, 0.5% Nonidet P-40) containing 10 mM β-glycerophosphate, 10 mM NaF, and 10 μg/mL leupeptin and aprotinin. Whole cell lysates were sonicated at 20% amplitude for 40 × 5 s in ice with 30 s cooling period between each burst. The lysate was centrifuged at 12,000 x g for 30 min at 4°C, and the recovered supernatant was mixed with 0.1 mL of 50% (v/v) prewashed M2 beads and incubated under gentle agitation in an end-over-end rotor at 4°C for 1 h. The beads were washed 3 times with 1 mL ice-cold NETN buffer and 3 times with 2 mL PBS. STK19-flag was eluted from M2 beads with 100 μl of elution buffer (1 mM flag peptide, 50 mM Tris-HCl, 10 mM MgCl2 and 1mM DTT) by gentle agitation for 2 h at 4°C. The beads were collected by centrifugation and the supernatant was transferred to a new Eppendorf tube. The protein concentration was determined, and the elution was analyzed on a 12% SDS-PAGE gel followed by Coomassie and silver staining. The final fraction was diluted to 0.5 μg/μL with wash buffer (50 mM Tris-HCl, pH 7.5, 10 mM MgCl2 and 1 mM DTT), aliquoted, snap-frozen with LN2 and stored at −80°C.

#### Kinase Assays

Kinase reactions contained 0.7 μg of highly purified NRAS bound to non-hydrolysable GTP (“active NRAS”), which was mixed with 2.5 μg of immunoprecipitated STK19-Flag, or with 0.25 μg of GST-STK19 (Signal Chem), or with increasing amounts of highly purified STK19 (70 ng, 240 ng, 700 ng and 1.4 μg respectively), essentially as described by Yin et al., 2019. Briefly, protein mixtures were incubated in a total of 20 μl kinase buffer (50 mM HEPES-NaOH, pH 7.9, 20 mM MnCl_2_) supplemented with 300 μM AMP, 100 μM ATP and 5 μCi of γ-32P-ATP and incubated for 30 min @ 30°C. After 30 min, 2.4 μl of 0.5 M EDTA (60 mM final) were added to quench the reaction and incubated for 5 min. Reactions were then stopped with 5x Laemmli sample buffer, boiled for 10 min at 70°C, loaded into a 10% Bis-Tris gel and ran with MES buffer @ 100–150 V. After electrophoresis, the radiolabelled gel was fixed in 40% ethanol, 10% acetic acid for 30 min, then stained using SilverQuest (Novex), following the manufacturer’s recommendations. After staining, the gel was rinsed with water, exposed overnight to a phosphorimager screen and then scanned using a Typhoon FLA 9500 (GE Healthcare).

### Quantification and Statistical Analysis

#### Analysis of RT-qPCR data

Biological triplicates (each in technical triplicate) were assayed for each condition, and the data were analyzed using multiple t tests with Holm-Sidak correction. Analysis details are also included in figure legends.

#### STK19 database peptide analysis and mass-spectrometry analysis of STK19 peptide intensities

For data base analysis, STK19 detected peptides were downloaded from peptide Atlas (Deutsch et al., 2008) and Proteomics DB (Schmidt et al., 2018) latest versions. To minimize unspecific mapping, peptides between 7 and 20, found in more than one experiment and with a maximum of one missed cut site were analyzed. Peptides with the same start or end position were merged for plotting. For the analysis of overexpressed mis-annotated STK19, eluted proteins from immunoprecipitations were separated by sodium dodecyl sulfate polyacrylamide gel electrophoresis (SDS-PAGE), until the running front had migrated approximately 1–2 cm into the gel (10% NuPAGE, Invitrogen, NP0301), and stained with colloidal Coomassie (InstantBlue, Expedeon). After excision of 8 horizontal gel slices per lane, proteins were in-gel digested with trypsin (Promega/Pierce) using a Janus liquid handling system (Perkin Elmer). Tryptic peptides were analyzed by liquid chromatography-mass spectrometry (LC–MS) using an Orbitrap Velos mass spectrometer coupled to an Ultimate 3000 uHPLC equipped with an EASY-Spray nanosource (Thermo Fisher Scientific) and acquired in data-dependent mode. The data were searched against the human Uniprot database using the Andromeda search engine. Raw data were processed using MaxQuant v1.6.0.1 (Cox and Mann, 2008). Peptide intensities were log2 transformed.

#### Human melanoma patient datasets

In order to analyze RNA-sequencing read coverage by exon (Figure 1E and Figure 4F/4G), we utilized raw RNA-seq data which we had available from four malignant melanoma cohorts: i) Van Allen et al. (Van Allen et al., 2015), ii) Snyder et al. (Snyder et al., 2014), iii) Hugo et al. (Hugo et al., 2016) and iv) a subset of the TCGA melanoma cohort with “D89N” mutation (from the total n = 8, raw data was available for n = 6). For cohorts with i)-iii), all cases with both RNA sequencing and whole exome (DNA) sequencing data were utilized (n = 81, total across the three cohorts). For gene level expression analysis (Figure 4E), where raw data was not required, we utilized processed RNA-seq expression data for melanoma patients from the cancer genome atlas (TCGA) project, obtained from the TCGA GDAC Firehose repository (https://gdac.broadinstitute.org/). Upper-quartile normalized count values from RSEM were utilized. Somatic *STK19* gene mutation calls for the same TCGA patient cohort were also obtained, from the cBioPortal. In total data from n = 473 patients were utilized, n = 465 wildtype (i.e., no D89N mutation) and n = 8 with D89N mutation.

#### Processing of raw RNA-seq data

Raw RNA-seq data was obtained in BAM format for all studies, and reverted back to FASTQ format using bam2fastq (v1.1.0). Sequence reads were aligned to hg19 genomic assembly, using bowtie pre-built index. Picard tools v1.107 was used to clean and sort the BAM files (http://broadinstitute.github.io/picard). Statistical analysis and figure plotting for the RNA-seq analyses were carried out using R3.5.1 (http://www.r-project.org/).

#### Splicing reads analysis

The Bioconductor package SGSeq [1] was used to construct a splice graph of the STK19 gene based on the Ensembl transcripts ENST00000466132 and ENST00000375331. The “analyzeFeatures” function was used to quantify the number of reads mapping to each exon and splice junction from available human melanoma patient dataset BAM files.

## Supplemental Information

Table S1 DNA and Oligos. Related to Key Resources Table and STAR Methods Sections, Quantitative PCR (qPCR), Generation of Stable Cell Lines, Plasmid Construction and Generation of STK19 (29 kDa) Baculovirus and Protein Expression
